# Prognostic value of programmed death-1, programmed death-ligand 1, programmed death-ligand 2 expression, and CD8(+) T cell density in primary tumors and metastatic lymph nodes from patients with stage T1-4N+M0 gastric adenocarcinoma

**DOI:** 10.1186/s40880-017-0226-3

**Published:** 2017-07-29

**Authors:** Yuan Gao, Su Li, Dazhi Xu, Shangxiang Chen, Yuchen Cai, Wenqi Jiang, Xinke Zhang, Jin Sun, Kefeng Wang, Boyang Chang, Fenghua Wang, Minghuang Hong

**Affiliations:** 1 Sun Yat-sen University Cancer Center; State Key Laboratory of Oncology in South China; Collaborative Innovation Center for Cancer Medicine, Guangzhou, 510060 Guangdong P. R. China; 2 Department of Clinical Trial Center, Sun Yat-sen University Cancer Center, Guangzhou, 510060 Guangdong P. R. China; 3 Department of Gastric Surgery, Sun Yat-sen University Cancer Center, Guangzhou, 510060 Guangdong P. R. China; 4 Department of Medical Oncology, Sun Yat-sen University Cancer Center, Guangzhou, 510060 Guangdong P. R. China; 5 Department of Pathology, Sun Yat-sen University Cancer Center, Guangzhou, 510060 Guangdong P. R. China; 6 Department of Image-guided Minimally Invasive Therapy, Sun Yat-sen University Cancer Center, Guangzhou, 510060 Guangdong P. R. China

**Keywords:** Gastric cancer, Programmed cell death-ligand 1, Programmed cell death-ligand 2, Programmed cell death-1, CD8(+) T cells, Heterogeneity expression, Prognostic value

## Abstract

**Background:**

Anti-programmed death-1/programmed death-ligand 1 (PD-1/PD-L1) immunotherapy has been proved to be effective on gastric cancer in ongoing clinical trials. However, the value of PD-L1 in predicting responses of patients with gastric cancer to anti-PD-1/PD-L1 immunotherapy is controversial. Some studies suggested that intra- and inter-tumoral heterogeneity of PD-L1 expression might explain the controversy. This study aimed to analyze the expression of PD-L1, PD-L2, and PD-1 as well as CD8(+) T-cell density in primary tumors and lymph nodes from patients with stage T1-4N+M0 gastric adenocarcinoma to explore the heterogeneity of PD-1 signaling pathway molecules.

**Methods:**

In primary tumors and metastatic as well as non-metastatic lymph nodes from patients with stage T1-4N+M0 gastric adenocarcinoma, we detected PD-L1 and PD-L2 expression with immunohistochemistry. CD8(+) T-cell density in primary tumors and PD-1 expression on CD8(+) T cells were detected with immunofluorescence. Univariate analysis was used to determine the prognostic values of them. Cox proportional hazard regression model was used to identify independent risk factors that affect patients’ overall survival and disease-free survival.

**Results:**

Among 119 eligible patients who had undergone surgical resection, the positive rate of PD-L1 was higher in metastatic lymph nodes than in primary tumors (45.4% vs. 38.7%, *P* = 0.005); the positive rate of PD-1 on CD8(+) T cells was significantly higher in primary tumors and metastatic lymph nodes than in tumor-free lymph nodes (both *P* < 0.001). The intensity of PD-1 expression on CD8(+) T cells in primary tumors and in metastatic lymph nodes were stronger than that in tumor-free lymph nodes from the same patient. Beside, the positive rate of PD-L2 did not show any differences between primary tumors and metastatic lymph nodes. In multivariate analysis, PD-L1 expression, PD-L2 expression, a low density of CD8(+) T cells in primary tumors, and PD-1 expression on CD8(+) T cells in primary tumors were associated with poor prognosis.

**Conclusion:**

The expression of PD-L1 is heterogeneous in primary tumors and in metastatic lymph nodes from patients with stage T1-4N+M0 gastric adenocarcinoma, which might explain the inconsistent results in assessing the prognostic value of PD-L1 expression in previous studies.

## Background

The programmed cell death-1 (PD-1)/programmed cell death-ligand 1 (PD-L1) pathway is important in the negative regulation of cell-mediated immune responses. Immunotherapy targeting the PD-1/PD-L1 axis has great promise in treating many types of cancer and was a breakthrough in cancer treatment. Exploring a molecular biomarker to predict clinical response to anti-PD-1/PD-L1 immunotherapy is thus critically important. However, there is no validated predictive biomarker that can identify patients who would likely respond to anti-PD-1/PD-L1 immunotherapy.

Immune checkpoint molecule PD-1 is expressed by various immune cells, including tumor-infiltrating CD8(+) T cells and CD4(+) T cells, and is activated by its ligands (either PD-L1 or PD-L2). PD-L1 and PD-L2 are expressed by antigen-presenting cells and cancer cells. PD-L1 expression has been detected in cancers of the skin [1], lung [2], breast [3], kidney [4], bladder [5], esophagus [6], stomach [7], head and neck [8], among others. PD-L1 expression, assessed with immunohistochemistry, is currently used in clinical trials as one potential biomarker to predict patients’ poor prognosis.

Some clinical trials have found that patients with PD-L1-positive tumors had higher rates of response to anti-PD-1/PD-L1 immunotherapy than patients with PD-L1-negative tumors [9–12], and others have found that patients with PD-L1-negative tumors also benefited from anti-PD-1/PD-L1 immunotherapy and that their objective response rate (ORR) was similar to that of patients with PD-L1-positive tumors [13, 14]. Questions arise when using immunohistochemical examination of PD-L1 expression as a companion diagnostic assay for anti-PD-1/PD-L1 immunotherapy. For example, how to determine the threshold that defines positive PD-L1 labeling; what impact the intra- and inter-tumoral heterogeneity of PD-L1 expression might take to the diagnostic assay; how to choose tissue compartment and immune cell population for detection of PD-L1 expression.

Gastric cancer (GC) is the fifth most common cancer globally, with a high incidence in East Asia, especially China [15–17]. Gastric adenocarcinoma is the most common type of GC. Many clinical, molecular, and pathologic data suggest that gastric adenocarcinoma is a heterogeneous disease [18]. Some studies have associated PD-L1 expression with the prognosis of gastric adenocarcinoma [7, 19, 20], although so far, research has focused only on primary tumors. Whether PD-L1 expression in gastric adenocarcinoma differs between primary tumors and metastatic sites is unknown. Moreover, several recent studies of anti-PD-1/PD-L1 antibodies in gastric adenocarcinoma have reported a relatively strong relationship between PD-L1 expression and the rate of response to anti-PD-1/PD-L1 immunotherapy [21–23], which makes it important to identify the exact expression condition of PD-L1 in patients with gastric adenocarcinoma.

We retrospectively detected the expression patterns of PD-L1 and PD-L2 in primary tumors and metastatic lymph nodes, the density of CD8(+) T cells in primary tumors, and the expression of PD-1 on CD8(+) T cells from patients with gastric adenocarcinoma, and determined their associations with clinicopathologic features and patient survival. To make sure the pathologic specimens of the primary tumor, metastatic lymph nodes, and tumor-free lymph nodes were available for every individual patient, we chose patients with stage T1-4N+M0 gastric adenocarcinoma.

## Patients and methods

### Patients

Patients with gastric adenocarcinoma who had undergone radical resection in Sun Yat-sen University Cancer Center (SYSUCC, Guangzhou, China) between January 2008 and December 2012 were selected in the study retrospectively. The inclusion criteria were as follows: (1) a diagnosis of gastric adenocarcinoma was confirmed by pathologic analysis; (2) the disease was classified as pathologic T1-4N+M0 cancer according to the 2010 American Joint Commission on Cancer TNM Staging Manual; (3) pathologic specimens were available for the primary tumor, metastatic lymph nodes, and tumor-free lymph nodes; (4) patients had completed at least 4 courses of adjuvant chemotherapy as documented in the medical record; and (5) complete follow-up data were available.

Tissue slides were constructed with materials collected from the Tissue Bank of SYSUCC, and this institution is allowed to perform translational research in compliance with ethical standards and patient confidentiality.

We had obtained consent from all patients to report their individual data. We also had obtained the approval from the Ethical Committee and Institutional Review Board of SYSUCC to conduct the present study.

### Immunohistochemistry

PD-L1 and PD-L2 expression was detected with immunohistochemistry in both primary tumors and metastatic lymph nodes. All lymph nodes were examined by pathologists after surgeries, and those with cancer cell invasion were conformed as metastatic lymph nodes. All metastatic lymph nodes were numbered and paraffin-embedded. Pathologists chose the metastatic lymph nodes with less necrosis for future detection. Formalin-fixed, paraffin-embedded tissue slides of 3-μm thickness were dewaxed in xylene and rehydrated through graded alcohol. To block endogenous peroxidase, the tissue slides were put in 3% H_2_O_2_ for 15 min. Tissue slides were then heated at 100 °C for 25 min in a microwave oven in EDTA (pH 8.0) (ThermoFisher Scientific, Waltham, MA, USA) for antigen retrieval. After cooling to room temperature, slides were incubated with 5% goat serum (ThermoFisher Scientific) for 60 min to prevent non-specific binding. Then, the slides were incubated with the primary antibodies of PD-L1 (E1L3N, 1:100, Cell Signaling Technology, Danvers, MA, USA) and PD-L2 (MAB1224, 1:200, R&D, Minneapolis, MN, USA) overnight at 4 °C. Afterward, the tissue slides were incubated with a goat anti-rabbit secondary antibody (Maixin Biotech, Fuzhou, Fujian, China) for 30 min. Then, 3,3-diaminobenzidine was used for color developing, and hematoxylin for nucleus counterstaining.

The expression of PD-L1 and PD-L2 was scored by two pathologists, both blinded to patients’ information. They selected five high-power fields per slide as representatives of the tumor, without known bias, and counted cancer cells with positive staining in these fields. The staining intensity was scored as follows: 0 for no staining, 1 for weak staining, 2 for moderate staining, and 3 for strong staining. The percentage of cancer cells with positive staining was scored as follows: 0%–5% positive staining was scored 0, 6%–25% scored 1, 26%–50% scored 2, 51%–75% scored 3, and 76%–100% scored 4 [19]. The final PD-L1 and PD-L2 expression score was calculated by multiplying the staining intensity score by the percentage score, the product of which ranged from 0 to 12. A receiver operating characteristic (ROC) curve and Youden Index (Youden Index = sensitivity + specificity − 1) were used to determine the optimal cut-off score of PD-L1 and PD-L2 expression. Specimens with an expression score higher than the cut-off were classified as positive; those equal to or lower than the cut-off were classified as negative.

### Immunofluorescence

The density of CD8(+) T cells in primary tumors and the expression of PD-1 on CD8(+) T cells in primary tumors, metastatic lymph nodes, and tumor-free lymph nodes were detected with immunofluorescence. The paraffin-embedded tissue slides were dewaxed and rehydrated, and antigen retrieval and non-specific binding prevention were performed as described in the previous subsection. The tissue slides were incubated with the primary antibody of CD8 (ab93278, 1:200, Abcam, Cambridge, Cambridgeshire, UK) and PD-1 (ab52587, 1:200, Abcam) overnight at 4 °C. The secondary antibodies (Alexa Fluor 488 goat anti-rabbit IgG [H + L] and Alexa Fluor 555 goat anti-mouse IgG [H + L], Life Technologies, Los Angeles, CA, USA) were used to bind the primary antibodies for 60 min at room temperature. After counterstaining with 4′,6-diamidino-2-phenylindole (DAPI) (P36931, Life technologies) for 5 min, the slides were observed under a confocal laser scanning microscope (OLYMPUS FV1000, OLYMPUS, Tokyo, Japan).

The density of CD8(+) T cells in primary tumors was scored as follows: 0 for no CD8(+) T-cell infiltration, 1 for focal infiltration with mostly perivascular distribution in tumor, 2 for moderate infiltration with prominent distribution among tumor cells, and 3 for severe infiltration with diffused distribution in tumor [24]. Scores of 2 and 3 were considered to indicate a high density of CD8(+) T cells infiltrating the tumor, whereas scores of 0 and 1 indicated a low density.

PD-1 expression on CD8(+) T cells was evaluated with fluorescence intensity as well as the percentage of CD8(+) T cells with positive PD-1 staining. The fluorescence intensity was scored from 0 to 3 for none, weak, moderate, and strong staining, respectively. The percentage of positive CD8(+) T cells was scored from 0 to 4 for 0%–5%, 6%–25%, 26%–50%, 50%–75%, and 76%–100% positive staining, respectively. The product of the two scores determined the final score of PD-1 expression, which ranged from 0 to 12. ROC and Youden Index were also used for PD-1 expression classification.

### Follow-up

The follow-up information was obtained from the Department of Follow-up at SYSUCC. The patients were followed via telephone every month. The final date of follow-up was June 30th, 2016.

### Statistical analysis

The associations between PD-L1 and PD-L2 expression in primary tumors and metastatic lymph nodes, the density of CD8(+) T cells in primary tumors, and PD-1 expression on CD8(+) T cells and clinicopathologic characteristics were evaluated with χ^2^ tests. Correlations between PD-L1, PD-L2, PD-1 expression and CD8(+) T-cell density were analyzed with the Kendall’s tau-b test. Overall survival (OS) was calculated from the date of diagnosis to the date of death. Disease-free survival was calculated from the date of diagnosis to the date of relapse. OS and DFS were analyzed with Kaplan–Meier curves. Patients alive at the end of the study were censored at the last follow-up. Variables with *P* values less than 0.05 in the univariate analysis were included in the Cox proportional hazards regression model for multivariate analysis.

All data were analyzed with the IBM SPSS Statistics 21 software (SPSS Inc., Chicago, IL, USA). Alpha was set at 0.05, and all tests were two-tailed.

## Results

### Patients’ characteristics

A total of 119 patients were eligible, with a median age of 55 years (range 25–66 years). The median number of courses of adjuvant chemotherapy was 6 (range 4–12). 85 patients were treated with the capecitabine–oxaliplatin or the leucovorin–fluorouraci–oxaliplatin regimen. The median follow-up was 28.0 months (range 4.5–92.0 months). As of the last follow-up visit, the cancer had progressed in 86 patients and was the cause of death in 80 patients.

### Cut-off score determination

**Fig. 1:**
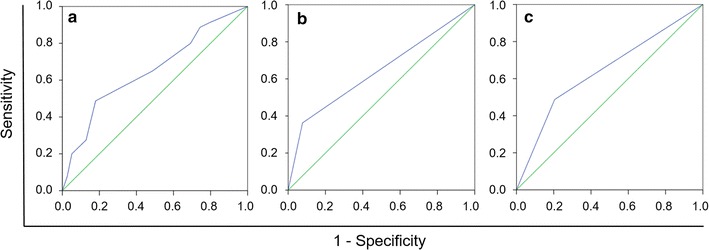
Determination of the cut-off scores for programmed cell death-ligand 1 (PD-L1) (**a**), programmed cell death-ligand 2 (PD-L2) (**b**), programmed cell death-1 (PD-1) (**c**) expression with the receiver operating characteristic (ROC) curve analysis

The areas under the ROC curves of PD-L1, PD-L2, and PD-1 expression were 0.652 [95% confidence interval (CI) 0.550–0.754, *P* = 0.007], 0.671 (95% CI 0.571–0.771, *P* = 0.003), and 0.627 (95% CI 0.527–0.726, *P* = 0.025), respectively (Fig. 1). A PD-L1 expression score of 4 maximized the Youden Index 0.163 as the optimal cut-off score. A PD-L2 expression score of 6 maximized the Youden Index 0.259 as the optimal cut-off score. A PD-1 expression score of 4 maximized the Youden Index 0.203 as the optimal cut-off score.

### PD-L1 expression

**Fig. 2:**
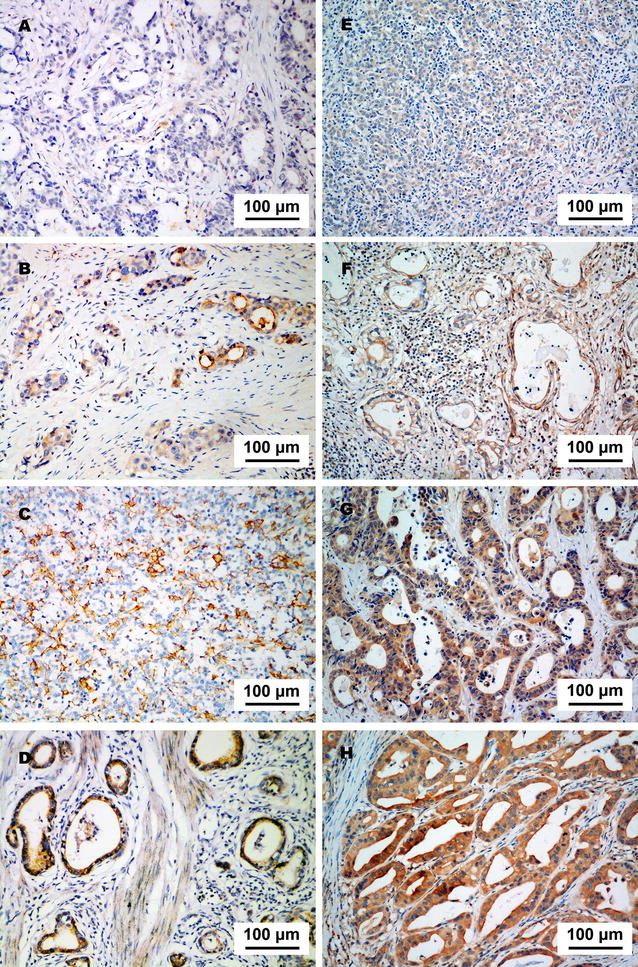
PD-L1 and PD-L2 expression in a specimen of gastric adenocarcinoma. **a** Negative staining of PD-L1. **b** Weak staining of PD-L1. **c** Moderate staining of PD-L1. **d** Strong staining of PD-L1. **e** Negative staining of PD-L2. **f** Weak staining of PD-L2. **g** Moderate staining of PD-L2. **h** Strong staining of PD-L2

**Fig. 3:**
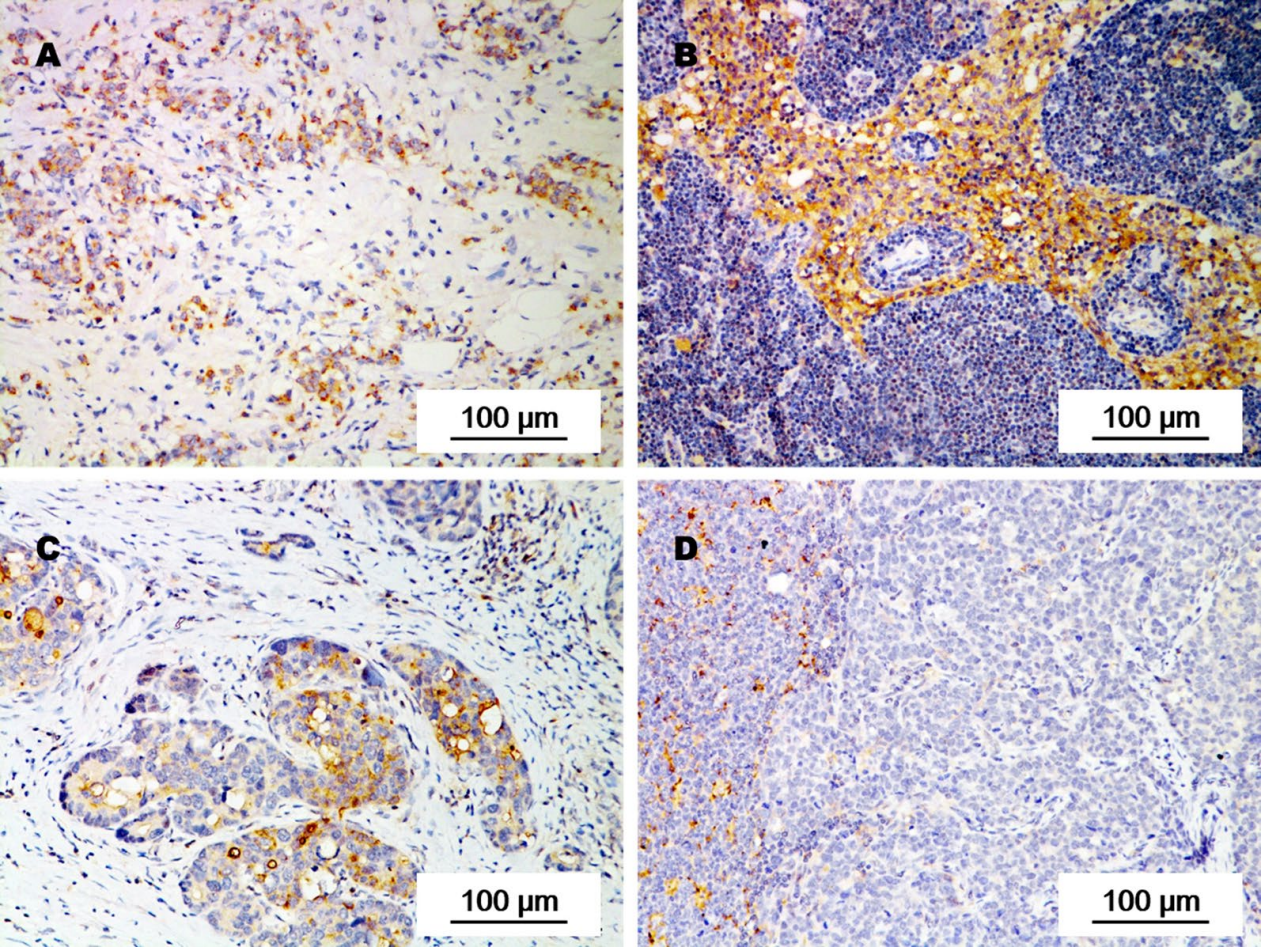
PD-L1 expression in primary tumors and corresponding metastatic lymph nodes from individual patients with gastric adenocarcinoma. PD-L1 expression was weaker in the primary tumor (**a**) than in metastatic lymph nodes (**b**) of a 71-year-old male patient with stage IIIB gastric adenocarcinoma. PD-L1 expression was stronger in the primary tumor (**c**) than in metastatic lymph nodes (**d**) of a 46-year-old female patient with stage IIIB gastric adenocarcinoma

PD-L1 was expressed predominantly in the cytoplasm and on the membrane of tumor cells (Fig. 2a–d). The positive rates of PD-L1 in both primary tumors and metastatic lymph nodes were significantly associated with Lauren classification and vascular invasion (all *P* < 0.05; Table 1). PD-L1 expression was inconsistent in primary tumors and metastatic lymph nodes in individual patients (Fig. 3). The positive rate of PD-L1 was significantly higher in metastatic lymph nodes than in primary tumors (45.4% vs. 38.7%, *P* = 0.005).

**Table 1:** Association between clinicopathologic factors and the expression of programmed cell death-ligand 1 (PD-L1) and programmed cell death-ligand 2 (PD-L2) in primary tumors and metastatic lymph nodes from patients with gastric adenocarcinoma

Variable	Total (cases)	PD-L1 expression [cases (%)]						PD-L2 expression [cases (%)]					
		Primary tumors			Metastatic lymph nodes			Primary tumors			Metastatic lymph nodes		
		Negative	Positive	*P*	Negative	Positive	*P*	Negative	Positive	*P*	Negative	Positive	*P*
Total	119	73	46		65	54		85	34		87	32	
Sex				0.102			0.352			0.904			0.991
Male	78	52 (66.7)	26 (33.3)		45 (57.7)	33 (42.3)		56 (71.8)	22 (28.2)		57 (73.1)	21 (26.9)	
Female	41	21 (51.2)	20 (48.8)		20 (48.8)	21 (51.2)		29 (70.7)	12 (29.3)		30 (73.2)	11 (26.8)	
Age (years)				0.781			0.450			0.911			0.122
<55	55	33 (60.0)	22 (40.0)		28 (50.9)	27 (49.1)		39 (70.9)	16 (29.1)		44 (80.0)	11 (20.0)	
≥55	64	40 (62.5)	24 (37.5)		37 (57.8)	27 (42.2)		46 (71.9)	18 (28.1)		43 (67.2)	21 (32.8)	
Tumor location				0.289			0.878			0.537			0.552
Cardia	49	34 (69.4)	15 (30.6)		28 (57.1)	21 (42.9)		37 (75.5)	12 (24.5)		38 (77.6)	11 (22.4)	
Body	20	12 (60.0)	8 (40.0)		11 (55.0)	9 (45.0)		15 (75.0)	5 (25.0)		15 (75.0)	5 (25)	
Antrum	50	27 (54.0)	23 (46.0)		26 (52.0)	24 (48.0)		33 (66.0)	17 (34.0)		34 (68.0)	16 (32.0)	
Cell differentiation				0.952			0.822			0.114			0.202
Well to moderate	21	13 (61.9)	8 (38.1)		11 (52.4)	10 (47.6)		12 (57.1)	9 (42.9)		13 (61.9)	8 (38.1)	
Poor (including Signet ring cell)	98	60 (61.2)	38 (38.8)		54 (55.1)	44 (44.1)		73 (74.5)	25 (25.5)		74 (75.5)	24 (24.5)	
Lauren classification				0.032			0.028			0.119			0.005
Intestinal	60	31 (51.7)	29 (48.3)		27 (45.0)	33 (55.0)		39 (65.0)	21 (35.0)		37 (61.7)	23 (38.3)	
Diffuse and mixed	59	42 (71.2)	17 (28.8)		38 (64.4)	21 (35.6)		46 (78.0)	13 (22.0)		50 (84.7)	9 (15.3)	
Vascular invasion				0.011			0.009			0.011			0.003
Yes	55	27 (49.1)	28 (50.9)		23 (41.8)	32 (58.2)		33 (60.0)	22 (40.0)		33 (60.0)	22 (40.0)	
No	64	46 (71.9)	18 (28.1)		42 (65.6)	22 (34.4)		52 (81.3)	12 (18.8)		54 (84.4)	10 (15.6)	
Neural invasion				0.381			0.679			0.001			0.004
Yes	77	45 (58.4)	32 (41.6)		41 (53.2)	36 (46.8)		63 (81.8)	14 (18.2)		63 (81.8)	14 (18.2)	
No	42	28 (66.7)	14 (33.3)		24 (57.1)	18 (42.9)		22 (52.4)	20 (47.6)		24 (57.1)	18 (42.9)	
Clinical stage				0.099			0.377			0.336			0.400
II–IIIA	12	10 (83.3)	2 (16.7)		8 (66.7)	4 (33.3)		10 (83.3)	2 (16.7)		10 (83.3)	2 (16.7)	
IIIB–IIIC	107	63 (58.9)	44 (41.1)		57 (53.3)	50 (46.7)		75 (70.1)	32 (29.9)		77 (72.0)	30 (28.0)

### PD-L2 expression

PD-L2 was also expressed predominantly in the cytoplasm and on the membrane of tumor cells (Fig. 2e–h). The positive rates of PD-L2 in both primary tumors and metastatic lymph nodes were associated with vascular invasion and neural invasion, and that in metastatic lymph nodes was associated with Lauren classification (all *P* < 0.05; Table 1). The positive rate of PD-L2 was not significantly different in primary tumors and metastatic lymph nodes (28.6% vs. 26.9%, *P* > 0.05).

### Density of CD8(+) T cells in primary tumors

The density of CD8(+) T cells in primary tumors was higher in non-intestinal type than in intestinal type gastric adenocarcinoma (*P* = 0.011). Stage IIIB–IIIC tumors exhibited a lower CD8(+) T-cell density than stage II–IIIA tumors (*P* = 0.018). The density of CD8(+) T cells in primary tumors was not associated with vascular invasion or neural invasion (Table 2).

**Table 2:** Associations between clinicopathologic factors and density of CD8(+) T cells and programmed cell death-1 (PD-1) expression on CD8(+) T cells in primary tumors and lymph nodes of gastric adenocarcinoma

Variable	Total (cases)	Density of CD8(+) T cells in primary tumors [cases (%)]			PD-1 expression on CD8(+) T cells [cases (%)]								
					Primary tumors			Metastatic lymph nodes			Tumor-free lymph nodes		
		Low	High	*P*	Negative	Positive	*P*	Negative	Positive	*P*	Negative	Positive	*P*
Total	119	46	73		74	45		72	47		106	13	
Sex				0.263			0.155			0.434			0.982
Male	78	33 (42.3)	45 (57.7)		45 (57.7)	33 (42.9)		45 (57.7)	33 (42.3)		69 (88.5)	9 (11.5)	
Female	41	13 (31.7)	28 (68.3)		29 (70.7)	12 (29.3)		27 (65.9)	14 (34.1)		37 (90.2)	4 (9.8)	
Age (years)				0.631			0.940			0.852			0.934
<55	55	20 (36.4)	35 (63.6)		34 (61.8)	21 (38.2)		34 (61.8)	21 (38.2)		49 (89.1)	6 (10.9)	
≥55	64	26 (40.6)	38 (59.4)		40 (62.5)	24 (37.5)		38 (59.4)	26 (40.6)		57 (89.1)	7 (10.9)	
Tumor location				0.782			0.464			0.552			0.648
Cardia	49	19 (38.8)	30 (61.2)		32 (65.3)	17 (34.7)		30 (61.2)	19 (38.8)		43 (87.8)	6 (12.2)	
Body	20	9 (45.0)	11 (55.0)		10 (50.0)	10 (50.0)		10 (50.0)	10 (50.0)		17 (85.0)	3 (15.0)	
Antrum	50	18 (36.0)	32 (64.0)		32 (64.0)	18 (36.0)		32 (64.0)	18 (36.0)		46 (92.0)	4 (8.0)	
Cell differentiation				0.660			0.981			0.799			0.906
Well to moderate	21	9 (42.9)	12 (57.1)		13 (61.9)	8 (38.1)		12 (57.1)	9 (42.9)		20 (95.2)	1 (4.8)	
Poor (including Signet ring cell)	98	37 (37.8)	61 (62.2)		61 (62.2)	37 (37.8)		60 (61.2)	38 (38.8)		86 (87.8)	12(12.2)	
Lauren classification				0.011			0.103			0.061			0.239
Intestinal	60	30 (50.0)	30 (50.0)		33 (55.0)	27 (45.0)		31 (51.7)	29 (48.3)		51 (85.0)	9 (15.0)	
Diffuse and mixed	59	16 (27.1)	43 (72.9)		41 (69.5)	18 (30.5)		41 (69.5)	18 (30.5)		55 (93.2)	4 (6.8)	
Vascular invasion				0.302			0.114			0.454			0.572
Yes	55	24 (43.6)	31 (56.4)		30 (54.5)	25 (45.5)		41 (64.1)	23 (35.9)		58 (90.6)	6 (9.4)	
No	64	22 (34.4)	42 (65.6)		44 (68.8)	20 (31.3)		31 (56.4)	24 (43.6)		48 (87.3)	7 (12.7)	
Neural invasion				0.488			0.457			0.433			0.951
Yes	77	28 (36.4)	49 (63.6)		46 (59.7)	31 (40.3)		23 (54.8)	19 (45.2)		38 (90.5)	4 (9.5)	
No	42	18 (42.9)	24 (57.1)		28 (66.7)	14 (33.3)		49 (53.6)	28 (36.4)		68 (88.3)	9 (11.7)	
Clinical stage				0.018			0.113			0.122			0.357
II–IIIA	12	1 (8.3)	11 (91.7)		10 (83.3)	2 (16.7)		10 (83.3)	2 (16.7)		11 (91.7)	1 (8.3)	
IIIB–IIIC	107	45 (42.1)	62 (57.9)		64 (59.8)	43 (40.2)		62 (57.9)	45 (42.1)		95 (88.8)	12 (11.2)	

### PD-1 expression

**Fig. 4:**
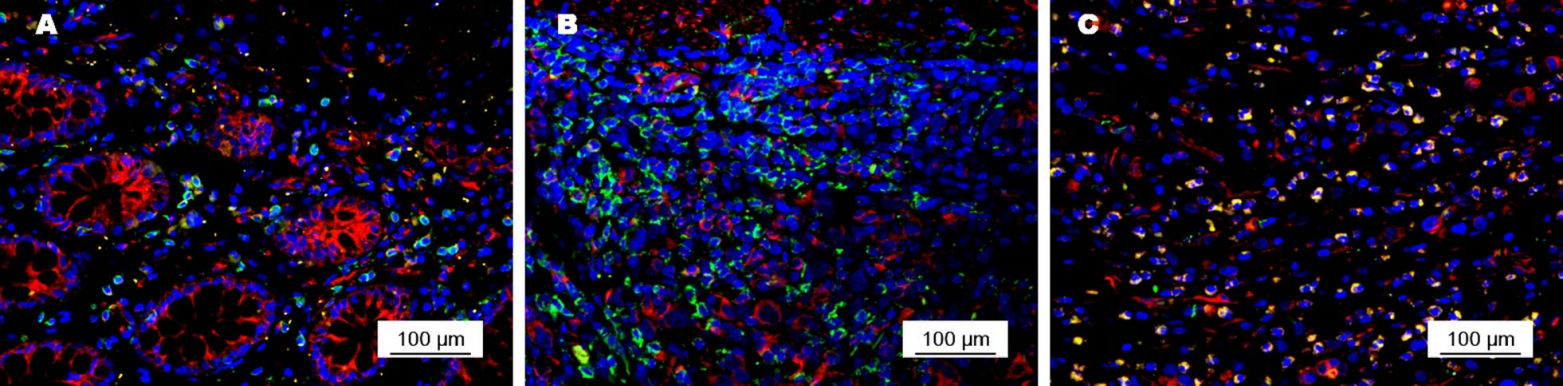
PD-1 expression (*red signals*) on CD8(+) T cells (*green signals*) in primary tumors of gastric adenocarcinoma. **a** Moderate intensity of PD-1 expression on CD8(+) T cells with a low density in the primary tumor; **b** weak intensity of PD-1 expression on CD8(+) T cells with a moderate density in the primary tumor; **c** strong intensity of PD-1 expression on CD8(+) T cells with a high density in the primary tumor

**Fig. 5:**
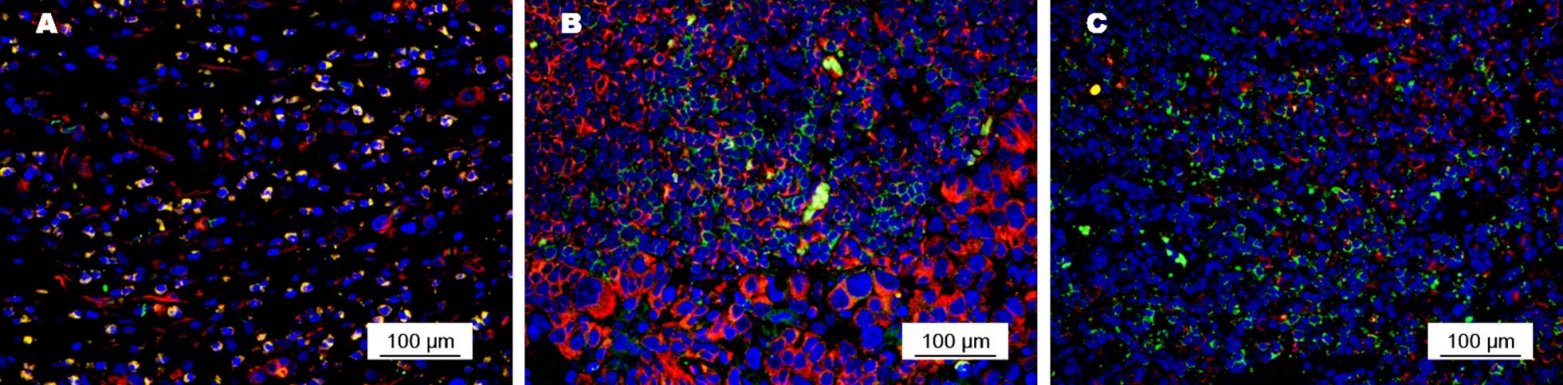
PD-1 expression (*red signals*) on CD8(+) T cells (*green signals*) in primary tumors and lymph nodes from a 54-year-old male patient with stage II gastric adenocarcinoma. PD-1 expression on CD8(+) T cells was stronger in the primary tumor (**a**) and in metastatic lymph nodes (**b**) than in tumor-free lymph nodes (**c**)

PD-1 was expressed predominantly on the membrane of tumor cells (Fig. 4). The positive rates of PD-1 expression on CD8(+) T cells were 37.8% in primary tumors, 39.5% in metastatic lymph nodes, and 10.9% in tumor-free lymph nodes. PD-1 expression on CD8(+) T cells in primary tumors was not associated with any clinicopathologic factors (Table 2). The intensity of PD-1 expression on CD8(+) T cells in primary tumors and in metastatic lymph nodes were stronger than that in tumor-free lymph nodes from the same patient (Fig. 5).

### Associations among PD-L1, PD-L2, PD-1 expression and CD8(+) T-cell density

PD-L1 expression was associated with PD-L2 expression in both primary tumors (*P* = 0.043) and metastatic lymph nodes (*P* = 0.012). In primary tumors, the density of CD8(+) T cells was associated with PD-L1 expression (*P* = 0.022) and PD-L2 expression (*P* = 0.004). Neither PD-L1 nor PD-L2 expression was associated with PD-1 expression on CD8(+) T cells in primary tumors (both *P* > 0.05; Table 3). In metastatic lymph nodes, both PD-L1 expression (*P* = 0.034) and PD-L2 expression (*P* = 0.002) were associated with PD-1 expression on CD8(+) T cells (Table 4).

**Table 3:** Associations among PD-L1, PD-L2, PD-1 expression, and CD8(+) T-cell density in primary tumors from patients with gastric adenocarcinoma

Variable	PD-L1 expression [cases (%)]			PD-L2 expression [cases (%)]		
	Negative	Positive	*P*	Negative	Positive	*P*
PD-L2 expression			0.043			
Negative	57 (47.9)	28 (23.5)				
Positive	16 (13.4)	18 (15.1)				
Density of CD8(+) T cells			0.022			0.004
Low	22 (18.5)	24 (20.2)		26 (21.8)	20 (16.8)	
High	51 (42.9)	22 (18.5)		59 (49.6)	14 (11.8)	
PD-1 expression on CD8(+) T cells			0.162			0.951
Negative	49 (41.2)	25 (21.0)		53 (44.5)	21 (17.6)	
Positive	24 (20.2)	21 (17.6)		32 (26.9)	13 (10.9)

**Table 4:** Associations among PD-L1, PD-L2, and PD-1 expression in metastatic lymph nodes from patients with gastric adenocarcinoma

Variable	PD-L1 expression [cases (%)]			PD-L2 expression [cases (%)]		
	Negative	Positive	*P*	Negative	Positive	*P*
PD-L2 expression			0.012			
Negative	54 (45.4)	11 (9.2)				
Positive	33 (27.7)	21 (17.6)				
PD-1 expression on CD8(+) T cells			0.034			0.002
Negative	45 (37.8)	27 (22.7)		60 (50.4)	12 (10.1)	
Positive	20 (16.8)	27 (22.7)		27 (22.7)	20 (16.8)	

### Univariate analysis on prognostic values of PD-L1, PD-L2, PD-1 expression and CD8(+) T-cell density

**Fig. 6:**
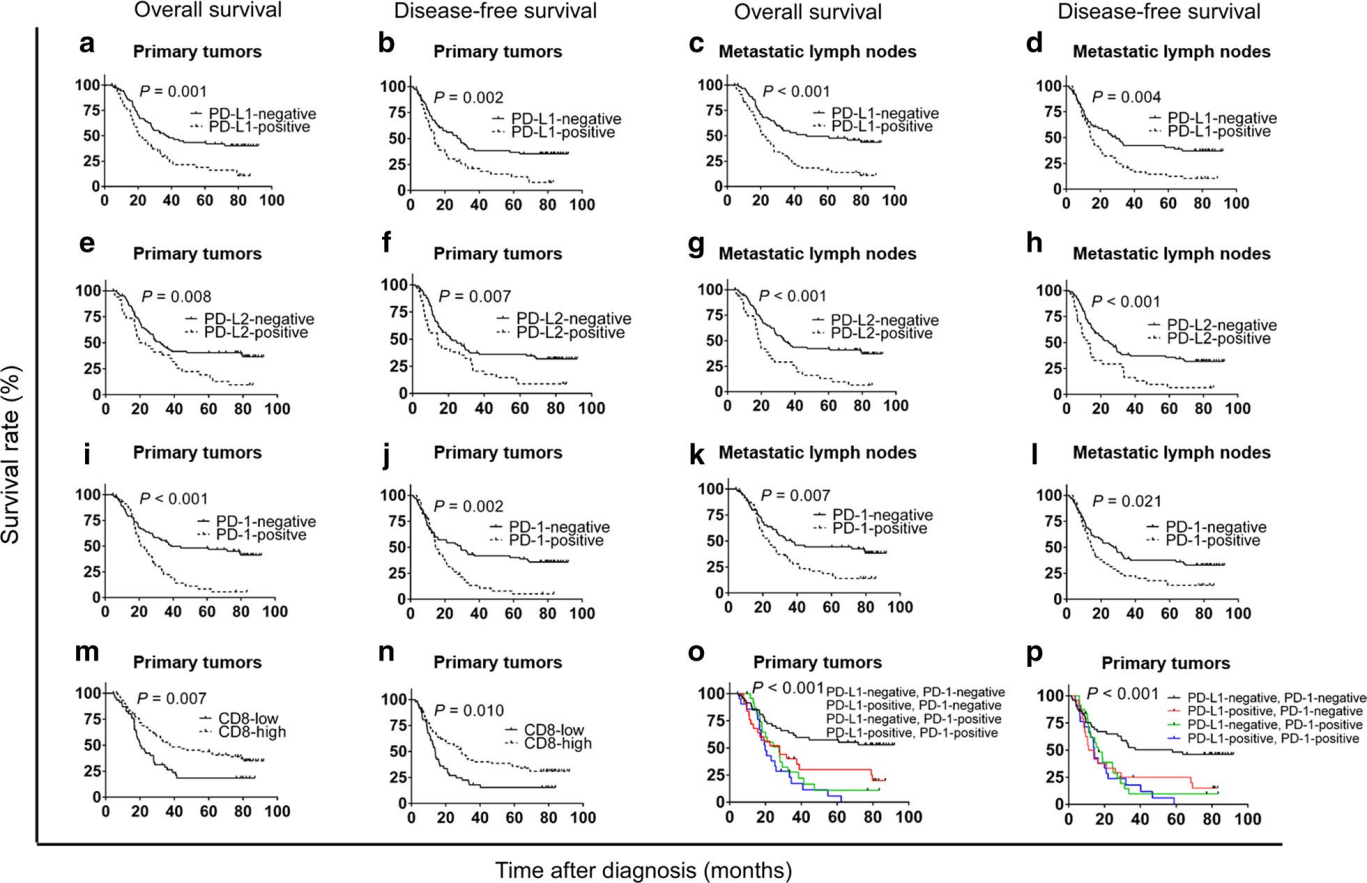
Overall survival (OS) and disease-free survival (DFS) curves of 119 patients with gastric adenocarcinoma according to PD-L1 expression, PD-L2 expression, CD8(+) T-cell density, and PD-1 expression on CD8(+) T cells. **a**, **b** OS and DFS curves according to PD-L1 expression status in primary tumors. **c**, **d** OS and DFS curves according to PD-L1 expression status in metastatic lymph nodes. **e**, **f** OS and DFS curves according to PD-L2 expression status in primary tumors. **g**, **h** OS and DFS curves according to PD-L2 expression status in metastatic lymph nodes. **i**, **j** OS and DFS curves according to PD-1 expression status on CD8(+) T cells in primary tumors. **k**, **l** OS and DFS curves according to PD-1 expression status on CD8(+) T cells in metastatic lymph nodes. **m**, **n** OS and DFS curves according to the density of CD8(+) T cells in primary tumors. **o**, **p** OS and DFS curves according to both PD-L1 and PD-1 expression in primary tumors

Patients with PD-L1 expression in primary tumors had lower 5-year OS and DFS rates than patients without PD-L1 expression (OS: 15.2% vs. 43.8%, *P* = 0.001, Fig. 6a; DFS: 10.9% vs. 38.4%, *P* = 0.002, Fig. 6b). Also, patients with PD-L1 expression in metastatic lymph nodes had lower 5-year OS and DFS rates than patients without PD-L1 expression (OS: 14.8% vs. 47.7%, *P* < 0.001; Fig. 6c; DFS: 13.0% vs. 40.0%, *P* = 0.004, Fig. 6d).

Patients with PD-L2 expression in primary tumors had lower 5-year OS and DFS rates than patients without PD-L2 expression (OS: 11.8% vs. 41.2%, *P* = 0.008, Fig. 6e; DFS: 8.8% vs. 35.3%, *P* = 0.007, Fig. 6f). Patients with PD-L2 expression in metastatic lymph nodes also had lower 5-year OS and DFS rates than patients without PD-L2 expression (OS: 9.4% vs. 41.4%, *P* < 0.001, Fig. 6g; DFS: 9.4% vs. 34.5%, *P* < 0.001, Fig. 6h).

Patients with PD-1 expression on CD8(+) T cells in primary tumors had lower 5-year OS and DFS rates than patients without PD-1 expression (OS: 17.0% vs. 43.1%, *P* < 0.001, Fig. 6i; DFS: 11.1% vs. 37.8%, *P* = 0.002, Fig. 6j). Patients with PD-1 expression in CD8(+) T cells in metastatic lymph nodes had lower 5-year OS and DFS rates than patients without PD-1 expression (OS: 17.0% vs. 43.1%, *P* = 0.007, Fig. 6k; DFS: 14.9% vs. 36.1%, *P* = 0.021, Fig. 6l).

Patients with a high density of CD8(+) T cells in primary tumors had higher 5-year OS and DFS rates than patients with a low density of CD8(+) T cells (OS: 39.7% vs. 21.7%, *P* = 0.007, Fig. 6m; DFS: 34.2% vs. 17.4%, *P* = 0.010, Fig. 6n).

When patients were allocated into four groups based on different combinations of PD-L1 expression in tumor cells and PD-1 expression on CD8(+) T cells in primary tumors, patients without PD-L1 and PD-1 expression had the best prognosis (Fig. 6o, p).

### Multivariate analysis on prognostic values of PD-L1, PD-L2, PD-1 expression and CD8(+) T-cell density

In multivariate analysis, PD-L1 expression in primary tumors, PD-L2 expression in metastatic lymph nodes, a high density of CD8(+) T cells in primary tumors, and PD-1 expression on CD8(+) T cells in primary tumors were independent prognostic factors for shorter DFS; PD-L1 expression in metastatic lymph nodes, PD-L2 expression in primary tumors, a high density of CD8(+) T cells in primary tumors, and PD-1 expression on CD8(+) T cells in primary tumors were independent prognostic factors for shorter OS. Besides, stage T3–T4 and tumor location were independent prognostic factors for shorter OS and DFS; neural invasion was an independent prognostic factor for shorter OS (Table 5).

**Table 5:** Multivariate analysis of prognostic factors for disease-free survival and overall survival of patients with gastric adenocarcinoma

Variable	Disease-free survival			Variable	Overall survival		
	HR	95% CI	*P*		HR	95% CI	*P*
T stage			<0.001	T stage			<0.001
T1–T2	1.000			T1–T2	1.000		
T3–T4	4.475	2.084–9.610		T3–T4	4.736	2.116–10.599	
Tumor location			0.005	Tumor location			0.002
Cardia	1.000			Cardia	1.000		
Body	1.327	0.691–2.547		Body	2.500	1.234–5.064	
Antrum	0.498	0.295–0.840		Antrum	0.671	0.394–1.143	
PD-L1 expression in primary tumors			0.025	Neural invasion			0.008
Negative	1.000			Negative	1.000		
Positive	1.735	1.073–2.807		Positive	2.172	1.220–3.459	
Density of CD8(+) T cells in primary tumors			0.009	PD-L2 expression in primary tumors			0.003
Low	1.000			Negative	1.000		
High	0.519	0.318–0.847		Positive	2.362	1.342–4.157	
PD-1 expression on CD8(+) T cells in primary tumors			0.014	Density of CD8(+) T cells in primary tumors			0.004
Negative	1.000			Low	1.000		
Positive	1.833	1.131–2.972		High	0.460	0.272–0.780	
PD-L2 expression in metastatic lymph nodes			0.033	PD-1 expression on CD8(+) T cells in primary tumors			0.006
Negative	1.000			Negative	1.000		
Positive	1.785	1.047–3.043		Positive	2.079	1.239–3.489	
				PD-L1 expression in metastatic lymph nodes			0.003
				Negative	1.000		
				Positive	2.198	1.301–3.712	

*HR* hazard ratio, *CI* confidence interval

## Discussion

We evaluated the expression of immune checkpoint molecules PD-L1 and PD-L2 in tumor cells, as well as the density of CD8(+) T cells and PD-1 expression on CD8(+) T cells in patients with T1-4N+M0 gastric adenocarcinoma. PD-L1, PD-L2, and PD-1 expression and a low density of CD8(+) T cells in primary tumors were associated with a poor prognosis.

The positive rate and prognostic value of PD-L1 expression in GC has been inconsistent. In one study that involved 102 patients with gastric adenocarcinoma in China, the positive rate of PD-L1 was 42.2% [25], which was slightly higher than that in our study. The difference in criteria to evaluate PD-L1 expression might account for the inconsistency. Besides, both studies found that PD-L1 expression in primary tumors was associated with vascular invasion and was an independent risk factor of prognosis. A study in Korea reported that PD-L1 expression was associated with vascular invasion and Lauren classification of gastric adenocarcinoma and was an independent risk factor of poor prognosis for patients with a high density of CD8(+) T cells in primary tumors [26], which were consistent with the results of our study. One study in USA also reported that PD-L1 expression in tumor cells from primary tumors was associated with poor prognosis of patients with gastric adenocarcinoma, but the positive rate of PD-L1 was only 12% [7], which was significantly lower than data from Asia studies [25, 26]. Although most studies showed that PD-L1 expression was associated with poor prognosis of GC, a Japanese study including 243 patients with curatively resected GC found that both PFS and OS were longer in PD-L1-positive patients than in PD-L1-negative patients [11]. It is possible that the difference in patients’ races might be related to the inconsistency.

The relationship between PD-L1 expression and patients’ response rate to anti-PD-1/PD-L1 immunotherapy was also not clear [27]. In the phase 1b KEYNOTE-012 study of 39 patients, pembrolizumab showed promising antitumor effect on PD-L1-positive, recurrent or metastatic gastric adenocarcinoma, with an ORR of 22% and a median OS of 11.4 months among these patients [21]. The prognostic and predictive values of PD-L1 expression in gastric adenocarcinoma patients who underwent anti-PD-1/PD-L1 immunotherapy thus remain to be explored. The inconsistent results from different studies might be explained by the different antibodies and specimens (frozen vs. paraffin-embedded tissues) used for detection [20]. In our study, we chose a commonly used, commercially available antibody E1L3N clone to detect PD-L1 expression. Besides the E1L3N clone, several other antibodies for PD-L1 expression detection are also used in clinical practice, including 9A11 (general usage), Ventana SP142 (for patients treated with atezolizumab), Ventana SP263 (for patients treated with durvalumab), Dako 22C3 (for patients treated with pembrolizumab), and Dako 28-8 (for patients treated with nivolumab). In a study assessing these six antibodies for the detection of PD-L1 expression with microarray immunofluorescent staining, concordance among four antibodies (SP142, E1L3N, 9A11, and SP263) revealed regression of tumor tissue cores (*R* ^2^ =  0.42–0.91) and cell line cores (*R* ^2^ =  0.83–0.97) [28]. All six antibodies had high levels of concordance (*R* ^2^ =  0.76–0.99) when using chromogenic staining in isogenic cell lines. A phase I trial involving 39 non-small cell lung cancer (NSCLC) patients compared four anti-PD-L1 antibodies (Dako 22C3, Dako 28-8, Ventana SP142, and Ventana SP263) using the Blueprint PD-L1 IHC Assay and demonstrated that the percentage of PD-L1-positive tumor cells was comparable when the 22C3, 28-8, and SP263 clones were used, whereas fewer tumor cells were stained with the SP142 clone [29]. Based on results from previous studies, the results of PD-L1 expression detected with the E1L3N clone in the present study was reliable and comparable with those of other studies.

Because PD-L1 expression can also be detected in stromal cells, the cell types chosen to detect PD-L1 expression might also influence the results [7]. We only detected PD-L1 expression in tumor cells in five high-power fields per slide which were selected without known bias to avoid influence caused by cell type.

We found significant heterogeneity of PD-L1 expression between primary tumors and metastatic lymph nodes. The abundant immune cytokines in the microenvironment of metastatic lymph nodes can induce PD-L1 expression, which might explain the increased positive rate of PD-L1 expression in metastatic lymph nodes than in primary tumors. However, some cases showed PD-L1 expression in the primary tumor, but not in metastatic lymph nodes. The heterogeneity of PD-L1 expression is probably responsible for the inaccuracy of using PD-L1 as a predictor of response to anti-PD-1/PD-L1 immunotherapy in current clinical trials. Sunshine and Taube [30] reported that patients with PD-L1 expression in pre-treatment specimens of primary tumors were more likely to respond to anti-PD-1/PD-L1 immunotherapy than those without PD-L1 expression. However, objective response was also observed in patients without PD-L1 expression, although with a relatively low rate [30]. Further exploration in one patient with several pre-treatment specimens found heterogeneous PD-L1 expression in the primary tumor, the metastatic lymph node, and subsequent subcutaneous metastases [31]. Thus, if PD-L1 expression status is detected in only one specimen, false negative results might reduce the efficiency of using PD-L1 as a predictor of response to anti-PD-1/PD-L1 immunotherapy.

Few studies have evaluated the expression and function of PD-L2 in patients with gastric adenocarcinoma. One study of anti-PD-1 immunotherapy for 41 patients with various cancer types reported that PD-L2 expression was detected in 8 patients with renal cell carcinoma, melanoma, or NSCLC [24]. Another study found a 51.7% rate of PD-L2 expression in patients with esophageal adenocarcinoma [32]. In our study, PD-L2 was expressed in 28.6% of patients with gastric adenocarcinoma and predicted short OS in those patients. As in another study, PD-L2 expression was positively correlated with PD-L1 expression in gastric adenocarcinoma, indicating a possible interaction between the two molecules [24].

The density of CD8(+) T cells in primary tumors has been reported to predict prognosis in various types of cancer [33–37]. In the present study, we found no difference in the positive rate of PD-L1 between patients with high and low CD8(+) T-cell density in primary tumors, in contrast to a USA study reporting that a high density of CD8(+) T cells was associated with a high positive rate of PD-L1 expression in gastric adenocarcinoma [7]. However, an Asian study on gastric adenocarcinoma also found no association between immunosuppressive proteins, such as PD-L1, cytotoxic T-lymphocyte-associated protein 4, and indoleamine 2,3-dioxygenase, and infiltration density of immune cells, including CD3(+), CD4(+), CD8(+), and PD-1(+) cells, in the tumor microenvironment [20]. This discrepancy might be explained by the mechanisms of PD-L1 overexpression in tumor cells and by the difference in signatures of tumor immunity between Asian and non-Asian patients with gastric adenocarcinoma. On one hand, PD-L1 could be up-regulated by both intrinsic aberrant pathways involved in carcinogenesis and extrinsic cytokines produced by other stromal cells in the tumor microenvironment [38–40]. On the other hand, the density of tumor-infiltrating T cells was lower in Asian patients with gastric adenocarcinoma than non-Asian patients as reported in a previous study [41], revealing a large distinction in the immune status of tumor environment in patients from different geographic areas. Besides, different characteristics of gastric adenocarcinoma between Asian and non-Asian populations might also be associated with the difference in median age of patients. The median age of patients in our study was 55 years old, which is consistent with the data reported by other studies in China [25, 42]. However, in the USA study, the median age of patients was 67 years old [7].

PD-1 is important in inhibiting the function of T cells by tumor cells through the PD-1/PD-L1 pathway [43], and it is a significant prognostic factor in several types of cancer [44–47]. PD-1 can be expressed on various types of activated T cells, and its function might be different [19]. The prognostic values of PD-1 expression on T cells were inconsistent among different studies [20, 46–48]. In the present study, we analyzed PD-1 expression on CD8(+) T cells and found it to be a unfavorable prognostic factor in gastric adenocarcinoma. The clinical value of PD-1 expression on other types of immune cells needs to be explored in further studies.

There were some limitations in the present study. First, we only analyzed the density of tumor-infiltrating CD8(+) T cells, but did not analyze the densities of CD3(+) T cells and CD4(+) T cells, which may also have prognostic values in GC patients. Second, the heterogeneity of PD-L1 expression was analyzed by comparing primary tumors with metastatic lymph nodes. If distant metastasis tissues could be obtained to detect PD-L1 expression, the heterogeneity analysis could be improved.

In conclusion, PD-L1 and PD-L2 expression in primary tumors and matched metastatic lymph nodes, low density of CD8(+) T cells in primary tumors, and PD-1 expression on CD8(+) T cells in primary tumors were unfavorable prognostic factors in patients with stage II/III gastric adenocarcinoma. PD-L1 expression was associated with PD-L2 expression as well as the density of CD8(+) T cells in primary tumors. PD-L1 expression in primary tumors was not consistent with that in metastatic lymph nodes. We suggest assessing multiple specimens when determining the status of PD-L1 expression in GC.

## Abbreviations

GC: gastric cancer
PD-L1: programmed cell death-ligand 1
PD-L2: programmed cell death-ligand 2
PD-1: programmed cell death-1
ROC curve: receiver operating characteristic curve
